# Reprogramming of Seed Metabolism Facilitates Pre-harvest Sprouting Resistance of Wheat

**DOI:** 10.1038/srep20593

**Published:** 2016-02-10

**Authors:** Caixiang Liu, Feng Ding, Fuhua Hao, Men Yu, Hehua Lei, Xiangyu Wu, Zhengxi Zhao, Hongxiang Guo, Jun Yin, Yulan Wang, Huiru Tang

**Affiliations:** 1 CAS Key Laboratory of Magnetic Resonance in Biological Systems, State Key Laboratory of Magnetic Resonance and Atomic and Molecular Physics, National Centre for Magnetic Resonance in Wuhan, Wuhan Institute of Physics and Mathematics, the Chinese Academy of Sciences, Wuhan 430071, China; 2 State Key Laboratory of Genetic Engineering, Collaborative Innovation Center for Genetics and Developmental Biology, Metabonomics and Systems Biology Laboratory, School of Life Sciences, Fudan University, Shanghai 200438, China; 3 Collaborative Innovation Center for Diagnosis and Treatment of Infectious Diseases, Zhejiang University, Hangzhou 310058, China; 4 College of Life Sciences, Wuhan University, Wuhan 430072, China; 5 Wuhan Zhongke Metaboss Ltd, 128 Guang-Gu-Qi-Lu, Wuhan 430074, China; 6 College of Plant Science and Technology, Huazhong Agricultural University, Wuhan 430070, China; 7 National Engineering Research Center for Wheat, Henan Agricultural University, Zhengzhou 450002, China

## Abstract

Pre-harvest sprouting (PHS) is a worldwide problem for wheat production and transgene antisense-thioredoxin-s (*anti-trx-s*) facilitates outstanding resistance. To understand the molecular details of PHS resistance, we analyzed the metabonomes of the transgenic and wild-type (control) wheat seeds at various stages using NMR and GC-FID/MS. 60 metabolites were dominant in these seeds including sugars, organic acids, amino acids, choline metabolites and fatty acids. At day-20 post-anthesis, only malate level in transgenic wheat differed significantly from that in controls whereas at day-30 post-anthesis, levels of amino acids and sucrose were significantly different between these two groups. For mature seeds, most metabolites in glycolysis, TCA cycle, choline metabolism, biosynthesis of proteins, nucleotides and fatty acids had significantly lower levels in transgenic seeds than in controls. After 30-days post-harvest ripening, most metabolites in transgenic seeds had higher levels than in controls including amino acids, sugars, organic acids, fatty acids, choline metabolites and NAD^+^. These indicated that *anti-trx-s* lowered overall metabolic activities of mature seeds eliminating pre-harvest sprouting potential. Post-harvest ripening reactivated the metabolic activities of transgenic seeds to restore their germination vigor. These findings provided essential molecular phenomic information for PHS resistance of *anti-trx-s* and a credible strategy for future developing PHS resistant crops.

Pre-harvest sprouting (PHS) is characterized by the premature germination of seeds occurring in the spikes and becomes a serious problem in many wheat-growing areas worldwide with prolonged pre-harvest rainfall and high humidity^1^. PHS leads to reduced yields, lower end-product quality of the grains and hence great economic losses. In China, for instance, about 25 million hectares of wheat are affected by PHS every year accounting for 83% of the whole wheat-growing area. Therefore, PHS resistant varieties are developed to solve the problem especially for the wheat-producing areas where long period of rainfall occurs frequently in the harvest season.

Pre-harvest sprouting is a complex phenotype resulting from interactions between wheat genotypes^2^ with biotic and/or abiotic environmental factors^3^. The untimely breakdown of seed dormancy is considered to be the major event for PHS to occur and therefore improvement of PHS resistance is often accompanied with prolonged seed dormancy to pass the harvest stage^4^.

Thioredoxin h (*trx-h*) gene is important for wheat seed germination and thus PHS^5^. *Trx-h* initially found in wheat kernels in 1979^6^ is now found widely present in the higher plants. This gene acts as an important regulator for seed germination by facilitating the reduction of intramolecular disulfide bonds in storage proteins of cereals, such as wheat and barley. During seed germination, *trx-h* also promotes the activation of α-amylase, pullulanase and proteases by weakening the inhibitive effect of inhibitor proteins on amylases and proteases^7^. Overexpressing *trx-h* gene in barley accelerated germination of the embryos and activated both α-amylase and starch pullulanase^8^,^9^. On the other hand, underexpressing *trx* h9 gene in wheat lowered the activities of Trx protein, α-amylase and pullulanase slowing seed germination^10^. It is particularly worth-noting that the transgenic wheat underexpressing *trx* h9 gene has also shown outstanding PHS resistance^10^.

*Trx-s* is another member of the thioredoxin gene family initially cloned from *Phalaris coerulescens*^5^. In fact, *trx-s* and *trx-h* have more than 90% homology in their cDNA sequences and similar biological functions for their expression products^11^. By using pollen-tube pathway, antisense thioredoxin s (*anti-trx-s*) was transferred into wheat to have successfully developed transgenic wheat with PHS resistance as well^12^. It was reported that *anti-trx-s* inhibited the endogenous *trx-h* expression and lowered α-amylase activity between day-30 post anthesis and 10 days post-harvest ripening resulting in high PHS resistance in the transgenic wheat^13^,^14^,^15^. It was also found that the introduction of *anti-trx-s* gene inactivated starch hydrolases and slowed hydrolysis of storage proteins^16^,^17^,^18^ in seeds imbibed for 3 to 4 days.

Systems biology approaches offer excellent opportunity to understand pre-harvest sprouting in terms of protein expressions and metabolism in a more holistic manner. Proteomic analyses already showed that transferred *anti-trx-s* gene caused down-regulation of many proteins in wheat seed kernels involving protein biosynthesis/degradation, starch degradation, gene expression regulation, lipid and energy metabolisms^19^. *Anti-trx-s* also caused up-regulation of proteins in kernels involving α-amylase activity suppression and disulfide bond formation compared to wild-type^19^. Several proteins related to stress resistance (such as antioxidant and disease resistance) were further up-regulated in the transgenic wheat kernels^19^. Moreover, transgenic wheat showed differential gene expression in *trx-h*, *serpin*, heat shock protein 70 (*hsp70*), and WRKY transcription factor 6 (*WRKY6*) compared to wild-type^19^. These results also imply that *anti-trx-s* gene may induce comprehensive metabolic changes in the transgenic wheat seeds. However, it remains unknown what metabolic changes such transgene causes, at which seed development stages and how these transgenic effects on seed metabolic activities are related to PHS.

Metabonomics ought to be a useful approach for understanding the dynamic metabolic changes since metabonomic analysis measures the metabolite composition (metabonome) of a given biological system and its dynamic responses to both endogenous and exogenous factors^20^,^21^,^22^. Such approach has been proven to be powerful in disease diagnosis^23^, in understanding metabolic variation between different rice varieties^24^ and metabolic responses to gene modifications^25^. Metabonomic analysis has increasingly become a powerful approach in understanding the effects of biotic and abiotic stressors on plant physiology and biochemistry^26^,^27^,^28^,^29^. So far, however, there have been no reports about the effects of *anti-trx-s* on the wheat seed metabonome, to the best of our knowledge, though these effects are expected to be insightful for developing PHS resistant wheat. It is also conceivable that PHS and its resistance ought to be associated with the development dependence of wheat seed metabolic phenome since sprouting of wheat seeds generally go through four grain filling periods including milk stage, dough development stage, mature seeds and post-harvest ripening period^15^.

In this study, we analyzed the seed metabonomic phenotypes (metabotypes) of transgenic wheat with *anti-trx-s* and wild-type at four different time-points of seed development (milk stage, dough development stage, mature seed and post-harvest ripeness period) using NMR spectroscopy in conjunction with multivariate statistical analysis. We also analyzed the developmental dependence of the fatty acid composition of these seeds using GC-FID/MS method. We further conducted integrative analysis on the metabonomic and proteomic differences between the PHS susceptible and resistant seeds. Our objectives are (1) to define the metabonomic changes induced by introduction of *anti-trx-s* and (2) to understand the molecular aspects of the PHS resistance acquired through introduction of such gene which will offer important information for further development of PHS-resistant wheat varieties.

## Results

Taking into consideration of the developmental periods of wheat seeds^15^, in this study, we analyzed metabonomic features of wheat seeds harvested at about day-20 post anthesis (20-dpa, milk stage), day-30 post anthesis (30-dpa, dough development stage), day-40 post anthesis (40-dpa, mature seed) and 30 days post-harvest ripening (30-dpr), respectively. We also considered the metabonomic data in an integrative manner with our proteomic results. Our results showed that this *anti-trx-s* transgenic wheat seeds had lower *trx-h* expression and α-amylase activity but higher PHS resistance compared with the corresponding wild-type wheat seeds as reported previously^13^,^14^,^15^. Our proteomic analysis results also showed that *anti-trx-s* gene introduction led to significant expressional changes of a number of metabolism-related proteins in wheat seeds^19^ at 40-dpa. This was highlighted by up-regulation of protein disulfide isomerase (PDI), serpin, xylanase inhibitor protein I precursor (XIP-I) but down-regulation of glucose-6-phosphate isomeraseprecursor (GPI), aldolase (ALD), glyceraldehyde-3-phosphate dehydrogenase (GAPDH), phosphoenolpyruvate carboxylase (PEPC), glutamate dehydrogenase (GDH), malate dehydrogenase (MDH), pullulanase, aspartate aminotransferase (AST), adenylylsulfate kinase (APK), α- and β-amylase^19^.

### Seed metabolite composition for transgenic and wild-type wheat

**Figure 1:**
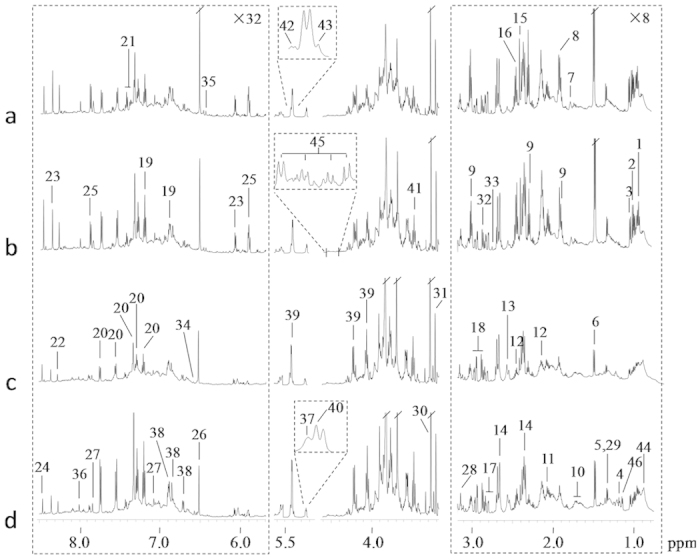
Average ^1^H NMR spectra of seed extracts from (**a**) transgenic wheat with *anti-trx-s* at 30-dpa, (**b**) wild type wheat at 30-dpa, (**c**) transgenic wheat with *anti-trx-s* at 40-dpa, and (**d**) wild type wheat at 40-dpa. The regions *δ* 0.75-3.19 and *δ* 5.68-8.50 were vertically expanded 8 times and 32 times, respectively. Keys: 1, Isoleucine (Ile); 2, Leucine (Leu); 3, Valine (Val); 4, Ethanol; 5, Threonine (Thr); 6, Alanine (Ala); 7, 4-Guanidinobutyrate (4-GB); 8, Acetate; 9, γ-Aminobutyrate (GABA); 10, Arginine (Arg); 11, Glutamate (Glu); 12, Glutamine (Gln); 13, Citrate; 14, Malate; 15, Succinate; 16, α-Ketoglutarate; 17, Aspartate (Asp); 18, Asparagine (Asn); 19, Tyrosine (Tyr); 20, Tryptophan (Trp); 21, Phenylalanine (Phe); 22, Adenosine triphosphate (ATP); 23, Adenosine; 24, Formate; 25, Uridine; 26, Fumarate; 27, Histidine (His); 28, Ethanolamine; 29, Lactate; 30, Betaine; 31, Choline; 32, Trimethylamine; 33, Dimethylamine; 34, *trans*-Aconitate; 35, Ferulate; 36, Guanosine; 37, D-Ribose-5-phosphate; 38, 4-Hydroxy-3-methoxyphenylacetate (HMPA); 39, Sucrose; 40, α-Glucose; 41, β-Glucose; 42, Raffinose; 43, Allantoin; 44, Lipids; 45, Nicotinamide adenine dinucleotide (NAD^+^); 46, Isobutyrate.

^1^H NMR spectra of seed extracts (Fig. 1) showed rich but obvious different metabolite profiles for *anti-trx-s* transgenic and wild-type wheat seeds at 30- and 40-dpa, respectively. Both ^1^H and ^13^C signals were unambiguously assigned to individual metabolites (Table S1) based on the literature data^30^,^31^,^32^, in-house databases and publicly available databases^33^ with further confirmation using a series of 2D NMR spectral data. More than 40 metabolites were identified including 16 amino acids and their metabolites (Ala, Thr, Val, Ile, Leu, Glu, Gln, Asp, Asn, Phe, Tyr, Trp, GABA, His, Arg and 4-guanidinobutyrate), 10 organic acids (*trans*-aconitate, citrate, α-ketoglutarate, succinate, malate, fumarate, formate, acetate, lactate, isobutyrate), 2 plant secondary metabolites namely 4-hydroxy-3-methoxyphenylacetate (HMPA) and ferulate, 6 nucleotide metabolites (uridine, adenosine, guanosine, allantoin, NAD^+^ and ATP), 5 choline metabolites (choline, ethanolamine, betaine, dimethylamine and trimethylamine), 4 sugars (sucrose, glucose, raffinose, D-ribose-5-phosphate) and ethanol (Fig. 1, Table S1 and Table 1). 15 fatty acids were also detected and quantified from these seeds (Table 2). Visual inspection showed that compared with wild-type, transgenic seeds had higher levels of fumarate and GABA at 30-dpa (Fig. 1a,b) but lower levels of Trp and Ala at 40-dpa (Fig. 1c,d). To obtain more detailed metabolic changes induced by *anti-trx-s* gene, multivariate data analyses were performed on the NMR data of these seeds.

### *Anti-trx-s* gene induced metabolic changes in transgenic wheat seeds

PCA (principal component analysis) scores plots showed clear development-associated metabonomic changes for both transgenic and wild-type wheat seeds reflected by a metabolic trajectory from 20-dpa, 30-dpa, 40-dpa and 30-dpr (Figure S1). Some obvious metabonomic differences were also observable between the transgenic and wild-type wheat seeds at 30-dpa, 40-dpa and 30-dpr (Figure S2B–D) although such difference appeared to be less prominent at 20-dpa (Figure S2A).

To obtain the detailed information on significant metabolic alterations induced by *anti-trx-s*, pairwise OPLS-DA (orthogonal projection to latent structure-discriminant analysis) was conducted between the extracts of transgenic wheat and wild-type seeds harvested at 20-dpa, 30-dpa, 40-dpa and 30-dpr, respectively; significantly differential metabolites between these two groups were tabulated in Table 1. At 20-dpa, OPLS-DA model parameters indicated only some limited metabolic differences were present between transgenic wheat and wild-type seeds. Careful analysis of all variables individually^34^ showed that at this development stage, only malate in the transgenic wheat seeds had significant level difference (*p* = 0.037) compared to the wild type (Table 1).

**Figure 2:**
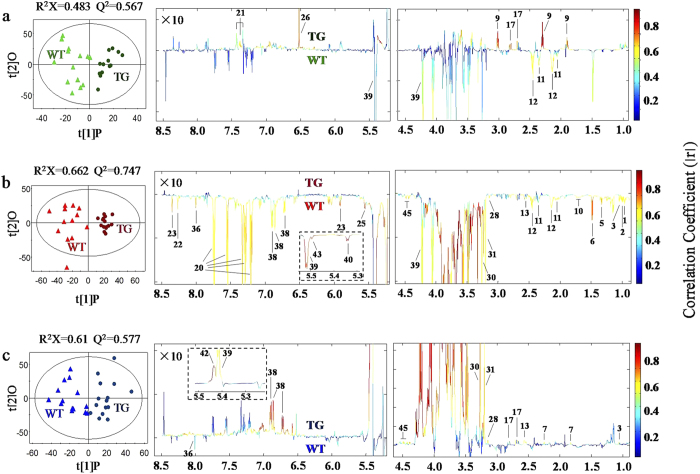
OPLS-DA scores plots (left) and coefficient-coded loadings plots (right) showing time-dependence of *anti-trx-s* gene effects on wheat seed metabolism. These models were derived from transgenic wheat (TG) and wild type (WT) seeds (**a**) at 30-dpa (*p* = 0.0007), (**b**) 40-dpa (*p* = 0.000001) and (**c**) 30-dpr (*p* = 0.0003). Metabolite keys are the same as in Fig. 1 and Table S1.

At the other three time points of seed development (i.e., 30-, 40-dpa and 30-dpr), in contrast, OPLS-DA model parameters (Fig. 2, Table 1) all showed significant seed metabonomic differences between the transgenic and wild-type wheat. The coefficient-coded loadings plots indicated that introduction of *anti-trx-s* caused significant elevation of seed fumarate, Asp, GABA and Phe at 30-dpa together with level decreases for sucrose, Glu and Gln compared with wild-type wheat (Fig. 2a, Table 1). At 40-dpa, *anti-trx-s* transgenic wheat seeds had significantly lower levels for 2 sugars (sucrose and glucose), 9 amino acids (Ala, Thr, Val, Ile, Leu, Glu, Gln, Trp and Arg), a TCA intermediate (citrate) and a secondary metabolite (HMPA), 3 choline metabolites (ethanolamine, choline and betaine) and 6 nucleotide derivatives (uridine, adenosine, guanosine, allantoin, NAD^+^ and ATP) than the wild type (Fig. 2b, Table 1). Furthermore, after 30 days post-harvest ripening (30-dpr), the transgenic wheat seeds had significantly lower level of guanosine but higher contents of sucrose, Val, citrate, HMPA, choline metabolites (ethanolamine, choline and betaine) and NAD^+^, being in contrast to the seed metabonomic features at 40-dpa. Moreover, introduction of *anti-trx-s* caused elevation of wheat seed raffinose, Asp and 4-guanidinobutyrate compared with the wild-type (Fig. 2c, Table 1).

**Figure 3:**
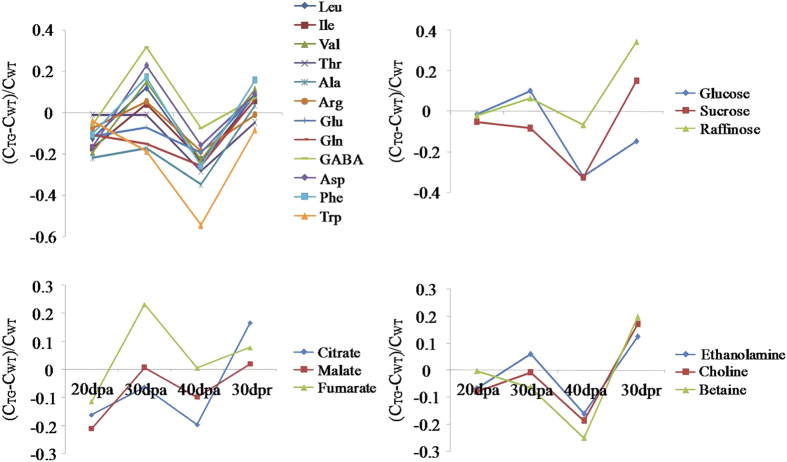
The ratios of concentration changes for metabolites in transgenic wheat seeds against that in wheat of wild type seeds. Metabolites with statistically significant variations are listed in Fig. 2 and Table 1.

We further calculated the ratios of concentration changes for the transgene-altered metabolites (against the wild-type wheat) at four different time points. The results indicated that compared with the wild type, a clear metabolic switch for the transgenic wheat occurred between 40-dpa and 30-dpr. At 40-dpa, for instance, the levels of Trp, Ala, Thr, Gln and Phe in the transgenic wheat seeds showed about 54%, 35%, 28%, 26% and 26% level decrease, respectively, compared with the wild-type (Fig. 3). In contrast, after 30 days post-harvest ripening, the levels of most seed amino acids in transgenic wheat were similar to these in wild type with exceptions for Asp and Val whose levels were higher in transgenic wheat (Fig. 3). Furthermore, much more significant differences between sucrose and glucose levels in transgenic wheat and wild-type were observed at 40-dpa than at the other three time-points; their levels were more than 30% lower in transgenic wheat seeds than in wild type (Fig. 3). After 30 days post-harvest ripening, in contrast, the levels of sucrose and raffinose were about 15% and 34% higher in transgenic wheat seeds than in wild-type (Fig. 3). Moreover, at-40 dpa, citrate and malate levels were lower in transgenic wheat seeds than wild type whereas, at 30-dpr, the levels of these metabolites and fumarate were higher in transgenic wheat seeds (Fig. 3). Ethanolamine, choline and betaine levels also showed a decrease at 40-dpa but an elevation at 30-dpr (Fig. 3) in transgenic wheat seeds compared with wild type.

### *Anti-trx-s* gene induced fatty acid changes in transgenic wheat seeds

Composition of wheat seed fatty acids was dominated by palmitate (C16:0), stearate (C18:0) and their unsaturated forms. Linoleic acid (C18:2n6) was the most abundant wheat seed fatty acid (34.1–46.1 μmol/g fresh weight) accounting for about 60% of total fatty acids (Table 2). Palmitic acid (C16:0, 11.7–15.0 μmol/g) and oleic acid (C18:1n9, 6.2–8.9 μmol/g) accounted for about 20% and 10% of total seed fatty acids, respectively. Linolenic acid (C18:3n3, 2.5–3.4 μmol/g) and stearic acid (C18:0, 1.2–1.4 μmol/g) were about 5% and 2% of total fatty acids, respectively.

Results showed outstanding dynamic changes in fatty acids induced by introduction of *anti-trx-s* with an obvious switch after 40-dpa. Compared with wild type seeds, the contents of total fatty acids (ToFAs), saturated fatty acids (SFAs), unsaturated fatty acids (UFAs), monounsaturated fatty acids (MUFAs), polyunsaturated fatty acids (PUFAs) and n6-type fatty acids were all decreased in transgenic wheat seeds at 40-dpa whereas the contrary was observed at 30-dpr (Table 2). Specifically, at 40 dpa, transgenic wheat seeds had significantly lower contents of C16:1n7 and PUFAs (C18:2n6, C18:3n3 and C20:4n6) than the wild-type (Table 2). At 30 dpr, in contrast, the concentration of C15:0, C16:0, C18:1n9, C20:1n9, C18:2n6 and C20:4n6 were significantly higher in transgenic wheat seeds than wild type (Table 2). Furthermore, at 40-dpa, the n6-to-n3 ratio for fatty acids was higher in transgenic wheat seeds than in wild type whereas the PUFA-to-MUFA ratio in transgenic wheat was lower than in wild type at 30-dpr (Table 2).

## Discussion

**Figure 4:**
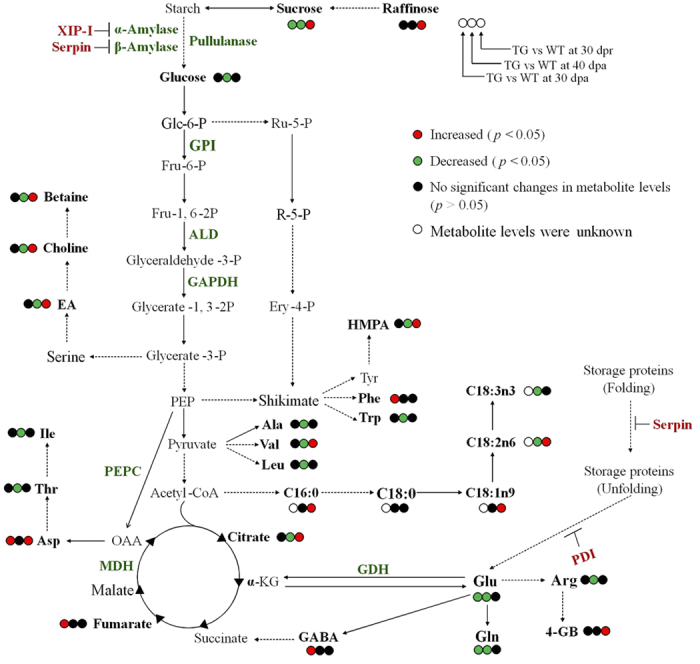
*Anti-trx-s* gene-induced metabolic changes in transgenic wheat compared with wild type wheat. Results for protein expressional changes were derived from Guo *et al.* (ref 19) at 40-dpa. Dark red bold letters and dark green bold letters denoted significant increases and decreases in protein levels respectively in transgenic wheat seeds compared with wild type. TG, transgenic wheat; WT, wild type wheat; 30-dpa, 30 days post anthesis; 40-dpa, 40 days post anthesis; 30-dpr, 30 days post-harvest ripening; Glc-6-P, glucose-6-phosphate; Fru-6-P, fructose-6-phosphate; Fru-1,6-2P, fructose-1,6-biphosphate; Ru-5-P, ribulose-5-phosphate; R-5-P, ribose-5-phosphate; Ery-4-P, erythrose-4-phosphate; Glyceraldehyde-3-P, Glyceraldehyde-3-phosphate; Glycerte-1,3-2P, Glycerte-1,3-biphosphate; Glycerte-3-P, Glycerte-3-phosphate; EA, ethanolamine; PEP, phosphoenolpyruvate; α-KG, α-ketoglutarate; HMPA, 4-hydroxy-3-methoxyphenylacetate; OAA, oxalacetate; GABA, γ-aminobutyrate; 4-GB, 4-guanidinobutyrate; XIP-1, xylanase inhibitor protein 1; GPI, glucose-6-phosphate isomerase; ALD, aldolase; PEPC, phosphoenolpyruvate carboxylase; GAPDH, glyceraldehyde-3-phosphate dehydrogenase; GDH, glutamate dehydrogenase; MDH, malate dehydrogenase; PDI, protein disulfide isomerase.

Wheat seeds with *anti-trx-s* gene expression showed excellent resistance to pre-harvest sprouting (PHS)^35^,^36^. The PHS resistant wheat differed significantly from the PHS susceptible wild-type in the seed metabonomic phenotypes at several seed development stages (Fig. 2 and Table 2). Such differences were outstanding especially when seeds reached maturity (at 40-dpa) and post-harvest ripening for 30 days (30-dpr). It is known that the period from day-5 pre-mature to day-10 post-mature is critical for wheat PHS to occur^15^. Our results showed that whilst most of the seed metabolic activities were inactivated at harvest time (40-dpa), these activities were restored after post-harvest ripening for a month (Fig. 4). This indicates that appropriate regulation and control of the seed metabolic activity during seed development is critical for pre-harvest sprouting to occur and thus for development of PHS resistant wheat.

This current study showed that introduction of *anti-trx-s* led to different metabonomic changes at different development stages in wheat seeds. In the early seed developmental stage (20-dpa or milk stage), only malate level in *anti-trx-s* transgenic wheat differed from that in wild type (Fig. 3). At this stage, the expression level of *trx-h* was only about 15.6% lower in the transgenic wheat than in wild type (Yumai-70)^15^. When *anti-trx-s* was transferred to another wild type cultivar Yangmai-5, no difference in α-amylase activity was observable between the transgenic and wild type wheat seeds at 20-dpa^37^. This implies that, at 20-dpa, the effects of introduction of *anti-trx-s* have only limited impacts on seed metabolism simply because the major metabolic events at this stage are programmed towards biosynthesis of materials needed for seed development. The transgenic effects on seed malate level may indicate that Krebs cycle was slower to some extent in the transgenic wheat during this early grain fill period.

When seeds reached dough development stage (at 30-dpa), much more metabolic differences were observable between the transgenic and wild type wheat seeds. GABA, Asp, Phe and fumarate were significantly up-regulated in the transgenic line whereas Glu, Gln and sucrose were significantly down-regulated (Fig. 2, Table 1). At this stage, about 23% decreases in the *trx-h* expression level were observed in the *anti-trx-s* transgenic wheat seeds compared with the control^15^. This indicated that biosynthesis of storage proteins and consumption of biogenic Glu was faster in transgenic wheat than in wild type offering nitrogen source for protein biosynthesis. Significant lower level of sucrose in transgenic seeds implied that *anti-trx-s* slowed the starch degradation to favor starch storage compared with wild type. At the dough development stage, therefore, the major metabolic events for both transgenic and wild type wheat seeds appeared to be biosynthesis of storage matters including starch and proteins although *anti-trx-s* expression seemed to accelerate such biosyntheses to some extent.

When wheat seeds reached maturity at 40-dpa, germinating ratio of transgenic wheat seeds was significantly lower than wild type^36^. Significant lower levels for 26 seed metabolites in the transgenic wheat than in wild type (Fig. 2, Tables 1,2) indicated comprehensive reduction of metabolic activities in *anti-trx-s* transgenic wheat seeds. This is agreeable with 36.5% decreases in α-amylase activity in *anti-trx-s* transgenic wheat seeds compared with the wild type control Yangmai-5 at 40-dpa^37^. These results are also consistent with the proteomic results for this wheat with significant up-regulations of XIP-I and PDI but down-regulations of GAPDH, GPI, ALD and PEPC^19^. This is reasonable since XIP-I suppresses α-amylase activity in wheat seeds^38^ whilst GAPDH, GPI and ALD are enzymes involved in glycolysis and thioredoxin can activate GAPDH^39^. Therefore, the markedly reduction of sucrose and glucose levels (Table 1, Fig. 2), XIP-I up-regulations and down-regulations of GAPDH, GPI and ALD^19^ suggests that both the seed starch degradation and glycolysis were slowed down in transgenic wheat at this particular stage (Fig. 4). PEPC catalyzing conversion of oxaloacetate into TCA intermediates can be hydrolyzed by enzymes having disulfide bonds^40^. At 40-dpa, expression of *anti-trx-s* gene in the transgenic wheat lowered thioredoxin h protein expression hence down-regulation of PEPC^19^. This and significantly lower citrate levels in the transgenic wheat seeds than in controls (Table 1) also suggested that TCA cycle was slowed down at 40-dpa in the transgenic wheat (Fig. 4).

Significant level decreases for many amino acids (Glu, Gln, Arg, Ala, Trp, Val, Leu, Ile and Thr) in transgenic wheat at 40-dpa suggest that expression of *anti-trx-s* gene affected protein metabolism. This and up-regulations of serpin and PDI^19^, two important proteins inhibiting breakdown of seed proteins, in transgenic wheat (Fig. 4) indicated that *anti-trx-s* expression slowed degradation of reserve proteins in seeds at this stage. Furthermore, down-regulations of GDH, AST and APK^19^ in the transgenic wheat (relative to wild type) indicated that *anti-trx-s* expression also slowed protein biosynthesis since these were essential enzymes for sulfydryl-dependent protein biosynthesis^41^,^42^.

Lower choline and ethanolamine levels in transgenic wheat seeds than in wild type are probably related to changes in membrane biosynthesis with choline and ethanolamine as the main fragments of membrane phospholipids in plants^43^. These two fragments can be produced through phospholipid hydrolysis catalyzed by phospholipase D (PLD). In fact, PLD antagonist did inhibit seed germination in *Arabidopsis*^44^. Our observation of choline and ethanolamine decreases resulting from introduction of *anti-trx-s* implies probable PLD inactivation in transgenic wheat to inhibit untimely seed germination (i.e., PHS) and/or down-regulation of glycerate-3-phosphate due to slowed glycolysis (Fig. 4). The decreases of adenosine, guanosine and uridine in transgenic wheat seeds were probably associated with the reduced overall metabolic activities of seeds including nucleic acid degradation and *de novo* biosynthesis purines and pyrimidines. The level reduction of allantoin further supports this notion with it as a catabolic metabolite of purines. Since wheat seed germination accompanies initiation of DNA and RNA biosynthesis^45^, it is reasonable that the decreased adenosine, guanosine and uridine levels in transgenic wheat seeds observed here are related to inhibition of potential seed germination. The change of HMPA associated with introduction of *anti-trx-s* indicates some roles of plant secondary metabolism in seed germination and such clearly warrants further investigations. Lower levels of main fatty acids in transgenic wheat seeds than in wild type (Table 2) are probably also related to slower overall metabolic activities in transgenic seeds although slowing glycolysis is one of the major reasons since *de novo* biosynthesis of fatty acids in plants is critically dependent on production of acetyl-CoA from glycolysis.

After 30 days postharvest ripening (30-dpr), 17 metabolites in *anti-trx-s* transgenic wheat seeds showed significant increase compared with wild type (Fig. 2, Tables 1,2) including NAD^+^, a TCA intermediate (citrate), two sugars (glucose, raffinose), three amino acid metabolites (Val, Asp, 4-guanidinobutyrate), three choline metabolites (choline, ethanolamine, betaine), a secondary metabolite (HMPA) and six fatty acids (C15:0, C16:0, C18:1n9, C18:2n6, C20:1n9 and C20:4n6). This indicates that break of seed dormancy occurs and transgenic wheat seeds start having more metabolic activities than corresponding wild type. Interestingly, 10 metabolites with lower levels in transgenic wheat at 40-dpa had higher levels at 30-dpr than the wild type controls (Tables 1,2). Nevertheless, at 30-dpr, the α-amylase activity together with the ratio and rates of germination were all similar for *anti-trx-s* transgenic and wild type seeds^36^,^37^ indicating both lines had similar starch degradation potentials required for germination. Compared with wild type, therefore, transgenic wheat seeds activate their metabolism to reduce the inhibiting effects of *anti-trx-s* gene so as to break dormancy and to initiate germination. Furthermore, 30 days postharvest ripening was sufficient for transgenic wheat seeds to recover the germination vigor by recovering the required metabolic activity of seeds.

To sum up, PHS-resistance of transgene *anti-trx-s* resulted largely from reprogramming seeds metabolism especially at later seed development (dough development, seed maturing) and post-harvest ripening stages. The expression of antisense thioredoxin s gene suppressed the overall metabolic activities of seeds when seeds reach maturity (40-dpa) (Fig. 4) so as to prevent from seed germination (i.e., pre-harvest sprouting). This metabolic reprogramming is critically important at about 40-dpa to prepare the seed metabolic activities since wheat seeds are most susceptible PHS at this stage^15^. During 30-days post-harvest ripening, however, the transgenic seeds re-adjusted their metabolic activities by up-regulating a number of pathways to overcome the *anti-trx-s* effects. This re-adjustment of metabolic activities is also critically important for seeds to break dormancy and germinate. Although *anti-trx-s* gene expressed throughout wheat growing period, its effects on seed metabolism was less obvious at milk (20-dpa) probably because biosyntheses of proteins, starch and nucleic acids were the dominant events in seed metabolism at such stage. These results offered essential biochemistry information for pre-harvest sprouting of wheat and for PHS resistance of anti-trx-s.

## Methods

### Plant materials

Seeds from the transgenic wheat with antisense *trx-s* gene and wild-type (Yumai 18) were harvested, respectively, at day-20 post anthesis (20-dpa, milk stage), day-30 post anthesis (30-dpa, dough development stage) and day-40 post anthesis (40-dpa) when seeds reached complete maturity. Wheat seeds harvested at 20-dpa and 30-dpa were snap-frozen in liquid nitrogen and stored at −80 °C until further analysis. Seeds at 40-dpa were divided into two groups with one group snap-frozen in liquid nitrogen followed with storage at −80 °C (designated as 40-dpa in this study). The other group of samples were kept at room temperature for 30 more days to have post-harvest ripening (and designated as 30-dpr here) followed with snap-frozen in liquid nitrogen and storage at −80 °C. Nine to eleven independent biological replicates were employed for 30-dpa, 40-dpa and 30-dpr whereas only six biological replicates were possible for samples harvested at 20-dpa.

### Seed metabolite extraction

Each sample was individually ground to fine power in liquid nitrogen with a mortar and a pestle followed with extraction with an optimized method for seeds^46^. In brief, above seed powder (150 ± 3 mg) was individually weighted into an Eppendorf tube followed with addition of 600 μL aqueous methanol (66%, v/v) and 3 mm tungsten carbide bead (Qiagen, Germany). The mixture was subjected to homogenized with a tissuelyser (Qiagen, Germany) followed with 15 min intermittent sonication in an ice bath (1 min sonication and 1 min break, repeated for 15 times). After centrifugation for 10 min (16099 × *g*, 4 °C), the supernatant was collected. This extraction process was further repeated twice and these three supernatants obtained were combined. After removal of methanol under vacuum, samples were lyophilized. The freeze-dried extracts were re-dissolved into 600 μL phosphate buffer (0.1 M, 50% D_2_O, pD 7.4) prepared from K_2_HPO_4_/NaH_2_PO_4_^47^ containing 0.002% TSP-*d*_*4*_. Following 10 min centrifugation (16099 × *g*, 4 °C), 550 μL of such solution from each sample was then transferred into a 5 mm NMR tube for metabolite analysis.

### NMR Measurements

All ^1^H NMR spectra were recorded at 298 K on a Bruker AVIII 600 spectrometer (600.13 MHz for ^1^H) equipped with an inverse detection cryogenic probe (Bruker Biospin, Germany). A standard noesypr1d pulse sequence (RD-90^o^-*t*_1_-90^o^-*t*_m_-90^o^-acquisition) was used to record one-dimensional ^1^H NMR spectra with the 90^o^ pulse length of about 10 *μ*s and t_1_ of 3 *μ*s. Water peak was saturated with a continuous wave irradiation during the recycle delay (RD, 2s) and mixing time (*t*_m_, 80 ms). A total of 64 transients were collected with 32 k data points over a spectral width of 12 kHz. For resonance assignment purposes, a set of 2D NMR spectra were recorded and processed as previously reported^48^,^49^ for selected samples including ^1^H-^1^H COSY, ^1^H-^1^H TOCSY, ^1^H-JRES, ^1^H-^13^C HSQC and ^1^H-^13^C HMBC spectra.

### Spectral processing and multivariate date analysis

An exponential line-broadening factor of 1 Hz was applied to each free induction decay (FID) prior to Fourier transformation (FT), and the spectra were phase- and baseline-corrected followed by referenced to TSP at *δ* 0.00. NMR spectral region between 0.5 and 8.5 ppm was divided into segments with width of 0.003 ppm using AMIX (v3.9.2, Bruker Biospin) whilst both methanol region at *δ* 3.339-3.384 and water region at *δ* 4.610-5.220 were removed.

The spectral areas of all buckets were normalized to the fresh weight of wheat seed power so that resultant data represented absolute metabolite concentration in the form of “peak intensity per weight unit of seeds”. PCA (principal component analysis) and OPLS-DA (orthogonal projection to latent structure-discriminant analysis)^50^ were carried out on the normalized NMR data using SIMCA-P+ software (v11.0, Umetrics, Sweden). In OPLS-DA models, one orthogonal and one predictive component were calculated using the unit-variance (UV) scaled NMR data as *X*-matrix and the class information as *Y*-matrix. The model qualities were described by the explained variances for *X-*matrix (R^2^X values) and the model predictability (Q^2^ values) with further assessment with CV-ANOVA approach^51^ where intergroup differences were considered as significant with *p* < 0.05.

The results were displayed in the forms of scores plots showing group clustering and loadings plots indicating variables (metabolites) contributing to inter-group differences. In these plots, loadings were back-transformed^52^ and the variables were color-coded according to the absolute values of the correlation coefficients (|r|)^53^ using an in-house developed script. In such plots, variable (i.e., metabolites) with hot colors (e.g., red) have more significant contributions to the group classification than the cold colored ones (e.g., blue). In this study, the metabolites exhibiting statistically significant changes were obtained at the level of *p* < 0.05. The ratios of changes for metabolites at four time-points were also calculated against their levels in wild-type wheat seeds in the form of (C_TG_ –C_WT_)/C_WT_, where C_TG_ stands for the metabolite concentrations in transgenic wheat seeds whereas C_WT_ stands for the metabolite concentrations in wild-type wheat seeds.

### GC-FID/MS analysis of fatty acids in the wheat seeds

Fatty acid composition in wheat seeds were measured in the methylated forms with a previously reported method^54^,^55^ with some minor modifications. About 20 mg of seed powder of wheat harvested at 40-dpa and 30-dpr was used with addition of 500 μL HPLC-grade CH_3_OH and one 3 mm tungsten carbide bead (Qiagen, Germany). Seeds at 20- and 30-dpa were not measured here due to limitation of samples availability. After vortex mixing, the mixture was homogenized with a tissuelyser (Qiagen, Germany) at 20 Hz for 90 s. Such treatment was repeated thrice with 3 min rest between each homogenization. 100 μL of homogenized mixture from each sample in a Pyrex tube was added with 20 μL internal standards in hexane (containing 1 mg/mL methyl heptadecanoate, 0.5 mg/mL methyl tricosanate and 2 mg/mL 3,5-di-tert-butyl-4-hydroxytoluene BHT) , then 1 mL mixture of methanol and hexane (4:l v/v). After cooling down the sample tubes above in a home-made liquid nitrogen bath for 10 min, 100 μL of precooled acetyl chloride was added carefully. Tubes were screw-capped and kept at room temperature in the dark for 24 hours followed with cooling down in an ice-bath for 4 min. 2.5 mL of 6% K_2_CO_3_ solution was then added (with gentle shaking) for neutralization. 200 μL hexane was added to extract the methylated fatty acids thrice and the combined supernatants were evaporated to dryness. The extracts were re-dissolved in 100 μL hexane for GC–FID/MS analysis.

Methylated fatty acids were quantified on a Shimadzu 2010 Plus GC-MS spectrometer. A flame ionization detector (FID) was used for quantification and a mass spectrometer with an electron impact (EI) ion source was employed for identification purpose. A DB-225 capillary GC column (10 m × 0.1 mm × 0.1 μm) was used with helium as carrier gas with 1 μL sample injected. The injection port and detector temperatures were set to 230 °C. The oven temperature was increased from 55 °C to 205 °C at 25 °C per min, kept at 205 °C for 3 min and then increased to 225 °C at 10 °C per min. The temperature was then kept at 225 °C for further 3 min.

All MS spectra over the m/z range of 45–450 were acquired with EI at 70 eV. Methylated fatty acids were identified by comparing retention times and mass spectra of 37 fatty acid standards. The results were expressed as micromole fatty acids per gram wheat seed fresh weight (FW).

### Proteomics analysis

Seed proteomics analysis for both transgenic and wild type wheat was done at 40-dpa as previously reported^19^.

## Additional Information

**How to cite this article**: Liu, C. *et al.* Reprogramming of Seed Metabolism Facilitates Pre-harvest Sprouting Resistance of Wheat. *Sci. Rep.* **6**, 20593; doi: 10.1038/srep20593 (2016).

## Supplementary Material

Supplementary Information

## Figures and Tables

**Table 1:** Significantly differential seed metabolites between *anti-trx-s* transgenic (TG) and wild type wheat (WT)^a^.

Metabolites (no.)	Coefficient (r)^b^		
	TG *vs*WT at 30-dpa	TG *vs* WT at 40-dpa	TG *vs* WT at 30-dpr
Amino acids & metabolites			
GABA (9)	0.77		
Asp (17)	0.66		0.53
Glu (11)	−0.57	−0.60	
Gln (12)	−0.57	−0.58	
Arg (10)		−0.57	
Ala (6)		−0.74	
Phe (21)	0.61		
Trp (20)		−0.70	
Val (3)		−0.61	0.59
Leu (2)		−0.58	
Ile (1)		−0.60	
Thr (5)		−0.70	
4-GB (7)			0.69
Sugars			
Sucrose (39)	−0.54	−0.90	0.66
Glucose (40, 41)		−0.92	
Raffinose (42)			0.88
TCA Cycle Metabolites			
Fumarate (26)	0.66		
Citrate (13)		−0.69	0.68
Nucleotide Metabolites			
Adenosine (23)		−0.74	
Uridine (25)		−0.77	
Guanosine (36)		−0.75	−0.54
Allantoin (43)		−0.86	
NAD^+^ (45)		−0.69	0.68
ATP (22)		−0.71	
Choline Metabolites and Others			
Ethanolamine (28)		−0.65	0.75
Choline (31)		−0.59	0.69
Betaine (30)		−0.65	0.79
HMPA (38)		−0.61	0.88

^a^The level of malate was lower in transgenic wheat than that in wild type seeds at 20-dpa (t-test, *p* = 0.037).

^b^The coefficients were obtained from OPLS-DA results, and positive and negative signs indicate positive and negative correlation in the concentrations, respectively.

**Table 2:** Seed fatty acid composition for wild type (WT) and transgenic (TG) wheat with *anti-trx-s* gene.

Fatty acids (μmol/g)	WT at 40-dpa	TG at 40-dpa	WT at 30-dpr	TG at 30-dpr
C14:0	0.08 ± 0.01	0.07 ± 0.01	0.08 ± 0.01	0.09 ± 0.10
C15:0	0.13 ± 0.01	0.12 ± 0.01	0.12 ± 0.02	0.16 ± 0.02*
C16:0	12.56 ± 0.72	11.72 ± 0.46	12.16 ± 0.67	14.99 ± 1.47**
C16:1n7	0.04 ± 0.01	0.02 ± 0.01**	0.04 ± 0.01	0.03 ± 0.01
C18:0	1.35 ± 0.10	1.21 ± 0.10	1.39 ± 0.10	1.30 ± 0.12
C18:1n9	7.01 ± 0.75	6.20 ± 0.82	6.32 ± 0.75	8.85 ± 0.97**
C18:2n6	40.09 ± 1.67	34.06 ± 3.18**	38.04 ± 2.45	46.05 ± 5.13*
C18:3n3	3.20 ± 0.13	2.54 ± 0.18**	2.98 ± 0.29	3.37 ± 0.45
C20:0	0.07 ± 0.01	0.06 ± 0.01	0.08 ± 0.01	0.07 ± 0.01
C20:1n9	0.32 ± 0.03	0.31 ± 0.04	0.28 ± 0.06	0.47 ± 0.08**
C20:2n6	0.06 ± 0.01	0.05 ± 0.01	0.06 ± 0.01	0.07 ± 0.01
C20:4n6	0.16 ± 0.01	0.13 ± 0.02*	0.12 ± 0.02	0.14 ± 0.01*
C22:0	0.09 ± 0.01	0.07 ± 0.01	0.08 ± 0.01	0.08 ± 0.01
C22:1n9	0.05 ± 0.01	0.06 ± 0.01	0.06 ± 0.01	0.08 ± 0.02
C24:0	0.10 ± 0.01	0.08 ± 0.01	0.10 ± 0.01	0.10 ± 0.01
Total FAs	65.3 ± 3.21	56.71 ± 4.66*	61.90 ± 4.06	75.87 ± 8.13**
SFAs	14.37 ± 0.75	13.35 ± 0.46*	14.01 ± 0.61	16.80 ± 1.60**
UFAs	50.93 ± 2.48	43.36 ± 4.23*	47.89 ± 3.46	59.06 ± 6.58**
MUFAs	7.42 ± 0.77	6.59 ± 0.86	6.70 ± 0.80	9.43 ± 1.07**
PUFAs	43.51 ± 1.78	36.77 ± 3.38**	41.19 ± 2.67	49.63 ± 5.57*
PUFA/MUFA	5.89 ± 0.40	5.60 ± 0.24	6.18 ± 0.34	5.27 ± 0.20**
PUFA/UFA	0.85 ± 0.01	0.85 ± 0.01	0.86 ± 0.01	0.84 ± 0.01
SFA%	22 ± 1	23 ± 1	23 ± 1	22 ± 1
UFA%	78 ± 1	76 ± 1	77 ± 1	78 ± 1
MUFA%	11 ± 1	12 ± 1	11 ± 1	12 ± 1
n3	3.20 ± 0.13	2.54 ± 0.18**	2.98 ± 0.29	3.37 ± 0.45
n6	40.31 ± 1.68	34.24 ± 3.20**	38.21 ± 2.46	46.26 ± 5.14*
n6/n3	12.59 ± 0.39	13.49 ± 0.38*	12.88 ± 0.87	13.76 ± 0.50

Data expressed as mean ± SD. *p < 0.05, **p < 0.01. SFAs, saturated fatty acids; MUFAs, monounsaturated fatty acids; PUFA, polyunsaturated fatty acids; n3, n3-type polyunsaturated fatty acids; n6, n6-type polyunsaturated fatty acids.
