# Supplementary material for: Reprogramming of Seed Metabolism Facilitates Pre-harvest Sprouting Resistance of Wheat
Source: Sci Rep. 2016 Feb 10;6:20593. doi: 10.1038/srep20593 (PMC4748292; doi:10.1038/srep20593)
Supplement: Supplementary Information [file srep20593-s1.pdf]

Supplementary information

Reprogramming of Seed Metabolism Facilitates Pre-harvest Sprouting Resistance of Wheat

Caixiang Liu[1], *Feng Ding*[4], *Fuhua Hao*[1], *Men Yu*[1,5], *Hehua Lei*[1], *Xiangyu Wu*[1], *Zhengxi Zhao*[6], *Hongxiang Guo*[7], *Jun Yin*[7], *Yulan Wang*[1,3,*] *and Huiru Tang*[1,2,*]

[1]CAS Key Laboratory of Magnetic Resonance in Biological Systems, State Key Laboratory of Magnetic Resonance and Atomic and Molecular Physics, National Centre for Magnetic Resonance in Wuhan, Wuhan Institute of Physics and Mathematics, the Chinese Academy of Sciences, Wuhan 430071, China

[2]State Key Laboratory of Genetic Engineering, Collaborative Innovation Center for Genetics and Developmental Biology, Metabonomics and Systems Biology Laboratory, School of Life Sciences, Fudan University, Shanghai 200438, China;

[3]Collaborative Innovation Center for Diagnosis and Treatment of Infectious Diseases, Zhejiang University, Hangzhou 310058, China

[4]College of Life Sciences, Wuhan University, Wuhan 430072, China

[5]Wuhan Zhongke Metaboss Ltd, 128 Guang-Gu-Qi-Lu, Wuhan 430074, China

[6]College of Plant Science and Technology, Huazhong Agricultural University, Wuhan 430070, China

[7]National Engineering Research Center for Wheat, Henan Agricultural University, Zhengzhou 450002, China

[1]To whom all correspondence should be addressed. Huiru Tang, Email, huiru_tang@fudan.edu.cn; tel, +86-21-51630725. Yulan Wang, Email, yulan.wang@wipm.ac.cn; tel, +86-27-87197143

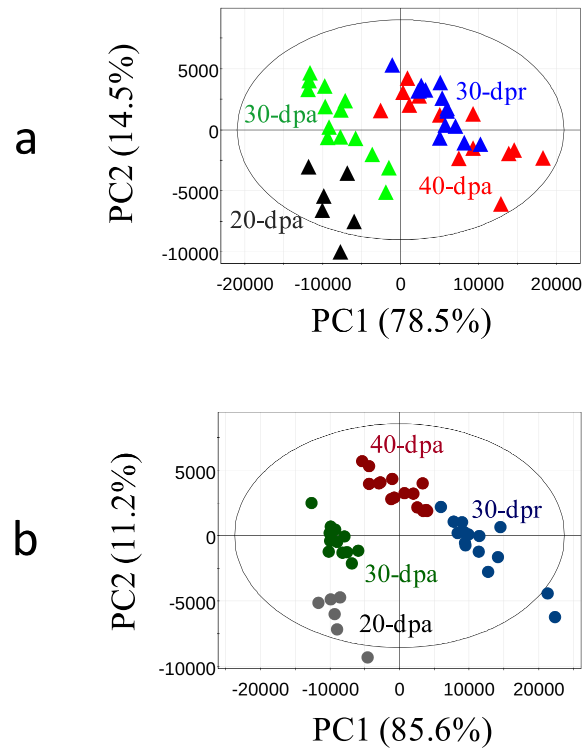

Figure S1. PCA scores plots for wild type (a) and *anti-trx-s* transgenic wheat (b) harvested at 20-dpa, 30-dpa, 40-dpa and 30-dpr respectively. The numbers in parentheses indicate the overall variance explained in the first two principal components.

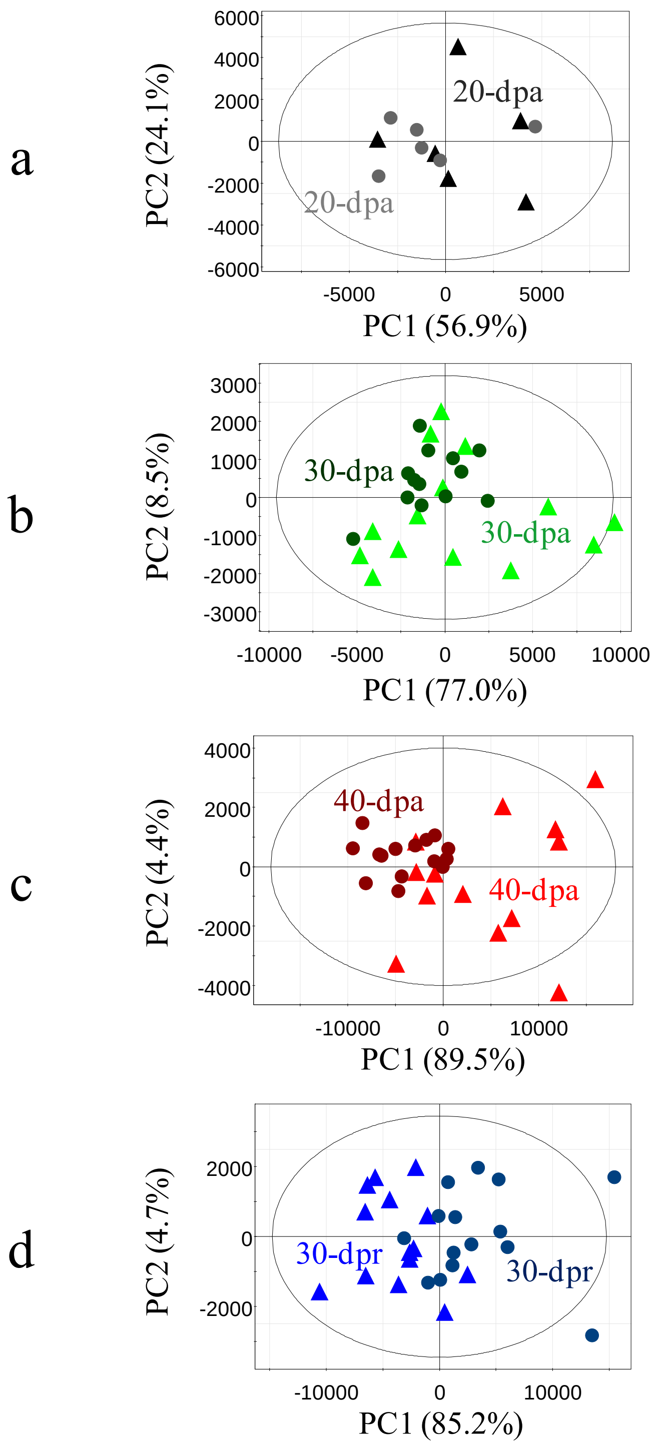

Figure S2. PCA scores plots for transgenic (dots) and wild type (triangles) wheat seeds at 20-dpa (a), 30-dpa (b), 40-dpa(c), and 30-dpr (d).

Table S1. Assignment of NMR data for metabolites in transgenic and wild type wheat seeds

no.	metabolites	moieties	δ^1H(ppm)	δ^{13}C (ppm)
1	Isoleucine (Ile)	δ-CH_3	0.94 (t)[a]	13.7
		γ-CH_3	1.01 (d)	17.5
		β-CH	1.97 (m)	38.7
2	Leucine (Leu)	δ'- CH_3	0.96 (d)	23.5
		δ- CH_3	0.97 (d)	24.8
		γ-CH	1.69 (m)	26.9
		β-CH_2	1.72 (m)	42.9
3	Valine (Val)	γ'- CH_3	0.99 (d)	19.8
		γ- CH_3	1.04 (d)	20.9
		β-CH	2.27 (m)	31.7
		α- CH	3.61 (d)	63.4
4	Ethanol	β-CH_3	1.18 (t)	19.8
		α-CH_2	3.66 (q)	60.6
5	Threonine (Thr)	γ- CH_3	1.33 (d)	20.8
		β-CH	4.24 (m)	68.6
		α- CH	3.57 (d)	63.4
		COOH		175.6
6	Alanine (Ala)	β- CH_3	1.48 (d)	17.9
		α-CH	3.78 (q)	53.4
		COOH		178.7
7	4-Guanidinobutyrate (4-GB)	γ- CH_3	3.19 (t)	42.9
		β-CH	1.83 (qu)	25.8
		α- CH	2.25 (t)	36.9
8	Acetate	α-CH_3	1.92 (s)	26.3
		COOH		184.1
9	γ-aminobutyrate (GABA)	γ- CH_2	3.02 (t)	42.3
		β- CH_2	1.91 (qu)	26.7
		α- CH_2	2.30 (t)	37.3
		COOH		184.2
10	Arginine (Arg)	δ- CH_2	3.23 (t)	43.3
		γ- CH_2	1.68 (m)	26.5
		β- CH_2	1.90 (c)	29.3
		α- CH	3.77 (t)	57.3
11	Glutamate (Glu)	γ- CH_2	2.36 (m)	34.7
		β- CH_2	2.02, 2.08 (m)	29.8
		α- CH	3.76 (t)	57.6
12	Glutamine (Gln)	γ- CH_2	2.45 (m)	32.3
		β- CH_2	2.14 (m)	27.5

		α- CH	3.76 (t)	55.1
		COOH		175.0,179.2.2
13	Citrate	α', γ'CH	2.66 (dd)	49.1
		α, γCH	2.55 (dd)	49.1
		COOH		180.1,184.5
14	Malate	β'-CH	2.37 (dd)	43.7
		β-CH	2.67 (dd)	43.3
		α-CH	4.31 (dd)	73.4
15	Succinate	α-CH_2	2.41 (s)	37.5
		COOH		184.6
16	α-Ketoglutarate (α-KG)	γ- CH_2	3.02 (t)	40.2
		β- CH_2	2.44 (t)	32.1
17	Asparate (Asp)	β'-CH	2.82 (dd)	37.6
		β-CH	2.67 (dd)	43.2
		α-CH	3.90 (dd)	53
		COOH		175.2,178.1
18	Asparagine (Asn)	β'-CH	2.96 (dd)	36.1
		β-CH	2.86 (dd)	35.9
		α-CH	4.01 (dd)	53.2
		COOH		174.5,174.2
19	Tyrosine (Tyr)	C2, 6H, ring	7.19 (d)	131.1
		C3, 5H, ring	6.90 (d)	115.4
		COOH		175.3
20	Tryptophan (Trp)	C7H, ring	7.55 (d)	111.1
		C6H, ring	7.29 (t)	121.1
		C5H, ring	7.20 (t)	118.5
		C4H, ring	7.74 (d)	117.6
		C2H, ring	7.33(s)	126
		COOH		175.1
21	Phenylalanine (Phe)	C2, 6, ring	7.33 (m)	124.3
		C3, 5, ring	7.43 (t)	127.5
		C4, ring	7.38 (m)	127.1
22	Adenosine triphosphate (ATP)	C5'H, ribose	4.28 (m)	68.2
		C2, ring	8.26 (s)	155.8
23	Adenosine	C1'H,ribose	6.06 (d)	92.3
		C8, ring	8.35 (s)	143.5
24	Formate	CH	8.46 (s)	169.3
25	Uridine	C1'H, ribose	5.90 (d)	104.5
		C6, ring	7.88 (d)	145.8
26	Fumarate	CH=CH	6.52 (s)	139.2
		COOH		177.5

27	Histidine (His)	5-CH	7.07 (s)	119.1
		3-CH	7.85 (s)	137.6
28	Ethanolamine (EA)	β- CH_2	3.83 (t)	56.3
		α- CH_2	3.15 (t)	42.6
29	Lactate	CH_3	1.33 (d)	22.5
		CH	4.12 (q)	71.5
		COOH		185
30	Betaine	N-(CH_3)+	3.27 (s)	54.1
		α- CH_2	3.90 (s)	66.6
31	Choline	N-(CH_3)$_3$	3.20 (s)	54.5
		N- CH_2	3.52 (m)	70.2
		O- CH_2	4.07 (m)	58.5
32	Trimethylamine	N- CH_3	2.88(s)	ND[b]
33	Dimethylamine	N- CH_3	2.72 (s)	ND
34	*Trans*-aconitate	CH_2	3.42 (s)	58.4
		CH	6.58 (s)	139.2
35	Ferulate	7-CH	7.33 (d)	128.8
		8-CH	6.42 (d)	124.3
36	Guanosine	8-CH	8.01 (s)	ND
37	D-Ribose-5-phosphate	1-CH	5.25 (d)	108.6
		4-CH	4.23 (d)	85.4
38	4-Hydroxy-3-methoxyphenylacetate	C2H, ring	6.89 (d)	119.4
	(HMPA)	C5H, ring	6.86 (d)	106.5
		C6H, ring	6.72 (dd)	112
39	Sucrose	Glc-C_1H	5.41 (d)	96.2
		Glc-C_2H	3.57 (dd)	72.1
		Glc-C_5H	3.83 (c)	63.3
		Fru- C_3H	4.22 (d)	77
		Fru- C_4H	4.06 (t)	75.1
		Fru- C_6H	3.83 (c)	63.2
40	α-Glucose	C_1H	5.24 (d)	95.9
		C_2H	3.55 (dd)	74.3
		C_4H	3.42 (dd)	72.8
41	β-Glucose	C_1H	4.65 (d)	98.7
		C_2H	3.25 (t)	77.3
		C_3H	3.49 (t)	78.9
		C_4H	3.41 (dd)	72.8
		C_6H	3.90 (dd)	64.3
42	Raffinose	Gal-C_1H	5.01 (d)	101.8
		Gal-C_4H	4.01 (d)	72.6
		Gal-C_5H	3.90 (m)	70.2

		Glc-C_1H	5.43 (d)	95.6
		Fru-C_3H	4.22 (t)	77.2
		Fru-C_4H	4.06 (t)	75.3
43	Allantoin	CH	5.39 (s)	66.1
44	Lipid	$CH_3(CH_2)$n-	0.89 (br)	
45	Nicotinamide adenine dinucleotide	α-CH_2	4.35 (m)	ND
	(NAD^+)	A3'CH	4.37 (m)	ND
		N3'CH	4.43 (m)	67
		N2'CH	4.49 (m)	71.1
		A2'CH	4.55 (m)	72.5
46	Isobutyrate	β-CH_3	1.15 (d)	20.4
		α-CH	2.36 (m)	ND

[a]Multiplicity: s, singlet; d, doublet; t, triplet; q, quartet; dd, doublet of doublets; qu, quintet; m, multiplet; c, complex; br, broad signals. [b]The signals were not determined.
